# Prediction of Liver Enzyme Elevation Using Supervised Machine Learning in Patients With Rheumatoid Arthritis on Treatment with Methotrexate

**DOI:** 10.7759/cureus.52110

**Published:** 2024-01-11

**Authors:** Sandeep Surendran, Mithun C B, Vinit Gilvaz, Prudhvi K Manyam, Kavya Panicker, Manu Pradeep

**Affiliations:** 1 Department of Rheumatology and Clinical Immunology, Amrita Institute of Medical Sciences, Amrita Vishwa Vidyapeetham, Kochi, IND; 2 Division of Rheumatology, Department of Internal Medicine, The Warren Alpert Medical School of Brown University, Providence, USA; 3 Department of Non-Communicable Disease Epidemiology, London School of Hygiene and Tropical Medicine, London, GBR

**Keywords:** medication safety, machine learning, transaminase elevation, methotrexate, rheumatoid arthritis

## Abstract

Objective

The aim of this study is to develop a machine learning (ML) model to accurately predict liver enzyme elevation in rheumatoid arthritis (RA) patients on treatment with methotrexate (MTX) using electronic health record (EHR) data from a real-world RA cohort.

Methods

Demographic, clinical, biochemical, and prescription information from 569 RA patients initiated on MTX were collected retrospectively. The primary outcome was the liver transaminase elevation above the upper limit of normal (40 IU/mL), following the initiation of MTX. The total dataset was randomly split into a training (80%) and test set (20%) and used to develop a random forest classifier model. The best model was selected after hyper-parameter tuning and fivefold cross-validation.

Results

A total of 104 (18.2%) patients developed elevated transaminase while on MTX therapy. The best-performing predictive model had an accuracy/F1 score of 0.87. The top 10 predictive features were then used to create a limited feature model that retained most of the predictive accuracy, with an accuracy/F1 score of 0.86. Baseline high-normal transaminase levels, and higher lymphocyte and neutrophil blood count proportions were the highest predictors of elevated transaminase levels after MTX therapy.

Conclusion

Our proof-of-concept study suggests the possibility of building a well-performing ML model to predict liver transaminase elevation in RA patients being treated with MTX. Similar ML models could be used to identify “high-risk” patients and target them for early stratification.

## Introduction

Methotrexate (MTX) is the most used disease-modifying anti-rheumatic drug (DMARD) in the treatment of rheumatoid arthritis (RA) [1]. Chronic use of this medication warrants close laboratory monitoring given its propensity for liver damage and myelosuppression [2]. MTX-associated hepatic dysfunction is a well-described adverse effect seen in up to a quarter of patients on long-term treatment [3]. The risk of hepatic dysfunction increases in patients with pre-existing liver damage, including non-alcoholic steatohepatitis (NASH), alcohol consumption, chronic viral hepatitis, and concurrent use of other hepatotoxic medications. Monitoring of therapy with frequent liver enzyme testing is recommended every two to three months for patients on a stable dose of MTX according to American College of Rheumatology (ACR) guidelines [4].

Recent advances in health care coupled with the extensive use of electronic health records (EHRs) have led to the accumulation of large amounts of patient data. Approaches such as machine learning (ML) allow us to leverage these large data sets to predict outcomes and support clinical decision-making [5]. There are two main types of ML algorithms or methodologies. The first is supervised ML, which aims to predict a known or predetermined outcome using labelled input data. Commonly used supervised ML algorithms are support vector machines and random forest algorithms. The other type is called unsupervised ML, where there are no outputs to predict; instead, the aim is to find naturally occurring patterns or groupings within the unsorted data using massive statistical computing power. Clustering and dimensionality reduction are typical examples of unsupervised learning [6].

Over the past decade, ML has been applied to various aspects of healthcare, and the field of rheumatology has been no exception. ML models have shown promise in various issues, ranging from automated detection of disease flares to predicting response to therapy [7-17]. One can note how ML has used diverse data sources to predict disease classification and genetic patterns and answer research questions accurately in rheumatology. ML modeling depends not only on the dataset size but also on the nature of the dataset. Overlap between the target variable and the dataset categories can result in bias and lead to overfitting. An overfitted ML model does well in testing but leads to inaccurate real-world applicability.

There is, however, a paucity of literature showing the application of ML for the prediction of therapy-related adverse events in rheumatology. The development of such models would allow the identification of high-risk patients who can be potentially targeted for closer monitoring to prevent these adverse outcomes. Conversely, low-risk patients can avoid extra outpatient visits, thereby reducing costs.

RA is the most common autoimmune inflammatory arthritis, with a global age-standardized point prevalence of 247 per 100,000 patients [18]. All clinical practice guidelines worldwide emphasize low-dose MTX as the cornerstone of treatment in RA [19]. Thus, developing a predictive model will help stratify and individualize long-term treatment for a sizable cohort of RA patients. To the best of our knowledge, no study has been published on predicting MTX-associated adverse events in RA using ML. With this background, we conducted this study using data from a real-world RA cohort. These data were then analyzed to develop a supervised ML model to predict the occurrence of liver enzyme elevation in RA patients on MTX for three months or longer.

## Materials and methods

Study design, participants, and settings

We conducted a retrospective cohort study, with ethical clearance obtained from the Institutional Ethics Committee (ECASM-AIMS-2023-507). Participant consent was waived as it was a retrospective analysis of routinely collected clinical data. We retrospectively reviewed the EHRs of all outpatient visits between April 2016 and September 2018 at the Department of Rheumatology of a quaternary care center in Kerala, India. Then, we included patients diagnosed with RA by a treating rheumatologist according to the 2010 ACR guidelines and were prescribed MTX. We also included seronegative patients with radiological features suggestive of RA. We used a step-up regimen at our center with patients being started on low-dose weekly MTX with or without bridge steroids and intra-articular injections on the first visit unless there were any absolute contraindications. The initial doses ranged from 10 to 15 mg per week and were titrated upward to 20-25 mg per week either singly or split daily dose according to the disease activity assessment. Patients who did not respond to monotherapy were hiked to combination DMARD therapy. The choice of the second agent depended on many factors such as patient preferences, cost, comorbid conditions, and the discretion of the treating rheumatologist. Patients who received biologics were excluded from the cohort. This was done to prevent selection bias in the data modelling, as patients are often shifted to biological therapy when they cannot tolerate full-dose MTX monotherapy, MTX and DMARD, or targeted synthetic DMARD (JAKi) combination therapy.

Data collection

From the EHRs, the following details were collected for the included participants: age, sex, the initial dose of MTX (mg/week), the maximum dose of MTX used (mg/week), and the duration of therapy (in years). In addition to the absolute doses, the initial and maximal doses were also grouped into <12.5 mg/week, >12.5 to <20 mg/week, and <20 mg/week for data analysis by the ML algorithm. The duration of therapy was also divided into more than 1.5 years and less than 1.5 years to ensure the clinical applicability of the model and avoid any risk of overfitting. The baseline transaminase levels (serum glutamate pyruvate transaminase[SGPT]/serum glutamic-oxaloacetic transaminase [SGOT]) and serum creatinine levels were also noted. We also collected treatment details of any concomitant use of leflunomide, sulfasalazine, mycophenolate mofetil, and hydroxychloroquine in combination or as a triple regimen. The DMARD prescriptions were classified according to different levels of the step-up protocol that individual patients were exposed to, i.e., patients on MTX monotherapy who had a flare-up and were hiked up to a combination DMARD therapy were included in both groups. This was because patients who are escalated to a combination therapy are often de-escalated to monotherapy over time. Also, the following baseline hemogram values were collected: hemoglobin (gm/dL), total white cell count, neutrophil and lymphocyte percentage, and neutrophil-lymphocyte ratio. The details about the presence of fatty liver on ultrasound at a grade of moderate/high were also collected from the electronic health system if they were available before the initiation of MTX. The primary outcome variable was an elevation of SGOT or SGPT more than the upper limits of the normal range at our reference laboratory (40 IU/L for both SGOT and SGPT). The tests were conducted using a Beckman-Coulter automated analyzer based on the spectrophotometry method in the central biochemistry laboratory of the quaternary center.

Statistical methods

All data from the electronic medical records were tabulated on MS Excel 2019 (Microsoft Corporation, Redmond, WA, USA) to perform descriptive analysis. Categorical data were presented as numbers and percentages. Continuous data were presented as means and standard deviations. Fisher’s exact and Pearson’s chi-square tests were used to test categorical data distribution, and odds ratio with 95% confidence interval was reported. Student’s t-test and Mann-Whitney U tests were used to compare continuous data.

Machine learning methodology

The data were imported to Python 3.0 using the JupyterLab (Project Jupyter, 2019) coding environment to apply the ML risk algorithms. The Scikit-learn Python library, specifically developed for data science and ML, was used for ML modeling. The development of hepatotoxicity was the target variable that was to be predicted. All the other 23 variables collected above were the inputs used. Missing values were considered absent/zero. This study cohort of RA patients (input) and the primary outcome (liver enzyme elevation) was split into training and validation cohorts. The training cohort was derived from a random sampling of 80% of the RA cohort, and the “validation” cohort comprised the remaining 20%. The “training” cohort data were imported into the Random Forest classifier algorithm. The random forest algorithm can be considered one of ML's representative algorithms and is known for its simplicity and effectiveness. It can also be defined as a decision tree-based classifier that chooses the best classification tree as the final classifier’s algorithm classification via voting. Decision trees work by learning simple decision rules extracted from the data features. The deeper the tree, the more complex the decision rules and the better the fit of the model attained [20].

**Figure 1:**
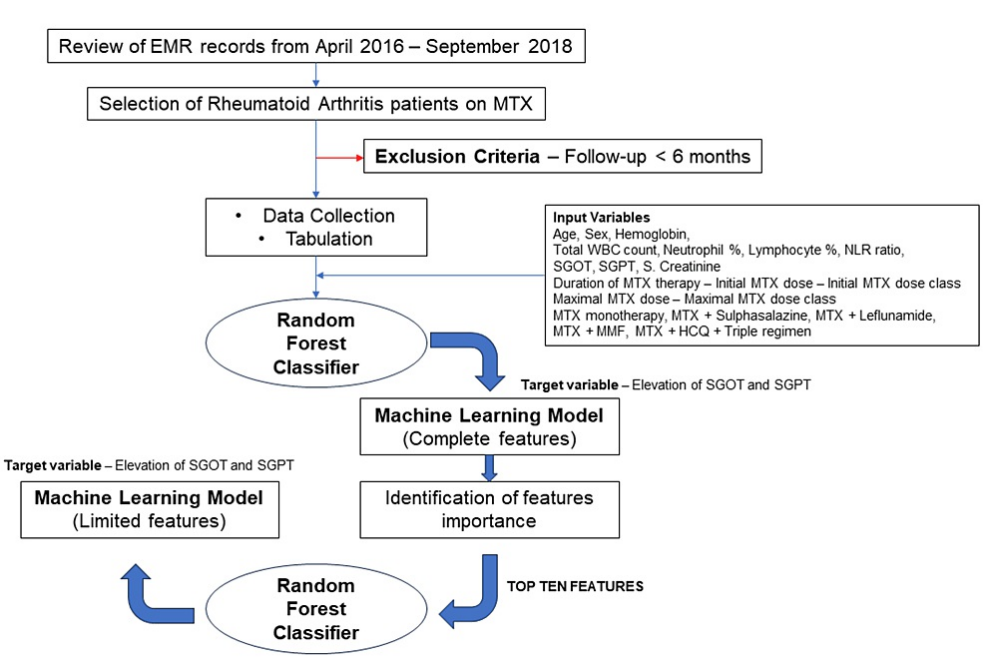
Development of the machine learning model from the RA cohort (full feature and limited feature) using the random forest classifier EMR, electronic medical records; MTX, methotrexate; SGOT, serum glutamic-oxaloacetic transaminase; SGPT, serum glutamate pyruvate transaminase; NLR, neutrophil-to-lymphocyte ratio; MMF, mycophenolate mofetil; HCQ, hydroxychloroquine

We also used a Python tool known as RandomisedSearchCV for hyper-parameter tuning to find the optimal parameters (Sklearn.Model_selection.RandomizedSearchCV, 2019). The best model developed using these 21 features was assessed using accuracy score, classification report (with precision, recall, and f-score), and receiver operating curve (ROC) plotting, along with the area under the curve (AUC) calculation. The feature importance of each variable in predicting liver enzyme elevation in the trained model were also calculated. Following this, the top 10 important features were then selected, and a limited features classifier model was trained using the same cohort, again split into training and validation sets (80% vs 20%). The performance of this limited features model was also assessed using precision, recall, and f-score, in addition to ROC plotting with AUC analysis. Figure 1 graphically shows the study methodology used to develop these classifiers (full and limited features).

## Results

Records of RA patients who visited the rheumatology outpatient clinic from September 1, 2016, to August 31, 2018, were examined for eligibility, and 569 patients were included for analysis from those. Among the included patients, 104 (18.2%) developed an elevation of liver enzymes while on MTX therapy. The baseline characteristics of the cohort stratified for the development of liver enzyme elevation are shown in Table 1.

**Table 1:** MTX-RA cohort stratified by the incidence of hepatic dysfunction (N = 569) NAFLD, non-alcoholic fatty liver disease; MTX, methotrexate; DMARD, disease-modifying anti-rheumatoid drugs; RA, rheumatoid arthritis

Baseline variables	Hepatic dysfunction, N (%)		OR (95% CI)	p-Value
	No (n= 465)	Yes (n=104)		
Age in years (mean ± SD)	52.33 ± 13.27	53.19 ± 10.61	-	0.194
Sex				
Male	64 (13.7%)	18 (17.3%)	1.3 (0.7–2.3)	0.352
Female	401 (86.3)	86 (82.7%)	(Reference)	
NAFLD on ultrasonography	5 (1.1%)	6 (5.7%)	5.6 (1.7–18.8)	0.005
MTX prescription patterns				
Initial MTX dose prescribed				
≤12.5 mg/week	321 (69.3%)	70 (67.3%)	(Reference)	0.821
>12.5 mg and <20 mg/week	131 (28.2%)	30 (28.8%)	(0.65–1.7)	
≥20 mg /week	13 (2.5%)	4 (3.8%)	1.4 (0.4–4.5)	
Maximal MTX dose prescribed				
≤12.5 mg/week	24 (5.1%)	3 (2.9%)	0.5 (0.2–1.8)	0.647
>12.5 mg and <20 mg/week	119 (25.6%)	26 (25.0%)	0.9 (0.6–1.5)	
≥20 mg/week	322 (69.3%)	75 (72.1%)	(Reference)	
Duration of MTX therapy of more than 1.5 years	200 (43%)	70 (67.3%)	2.7 (1.7–4.3)	< 0.001
Pattern of combination drug therapy with MTX				
MTX monotherapy	454 (97.6%)	104 (100%)		1.00
MTX plus sulfasalazine	48 (10.3%)	22 (21.2%)	2.3 (1.3–4.1)	0.002
MTX plus leflunomide	172 (37.0%)	41 (39.4%)	1.1 (0.7–1.7)	0.643
MTX plus mycophenolate mofetil	6 (1.3%)	1 (0.9%)	0.7 (0.1–6.2)	0.784
MTX plus hydroxychloroquine	208 (44.7%)	41 (39.4%)	0.8 (0.5–1.2)	0.324
MTX plus >1 DMARD (triple regimen)	10 (2.2%)	3 (2.9%)	1.3 (0.4–5)	0.714

Using the SciKit Random Classifier algorithm, an ML model was derived. The best model was selected after hyper-parameter tuning and fivefold cross-validation). A precision of 0.89, recall of 0.87, and an accuracy/F1 score of 0.87 were achieved. The feature importance of the input variables are shown in Table 2.

**Table 2:** Feature importance of the full-feature ML classifier model for MTX-associated hepatic dysfunction (N = 569) *Duration of MTX is divided into two groups: more or less than 1.5 years. NAFLD, non-alcoholic fatty liver disease; NLR, neutrophil-to-lymphocyte ratio; SGOT, serum glutamic-oxaloacetic transaminase; SGPT, serum glutamate pyruvate transaminase; MTX, methotrexate; ML, machine learning

Input variable	Feature importance
Age	0.073498
Sex	0.006436
NAFLD	0
Hemoglobin	0.070145
Total count	0.062881
Neutrophil percentage	0.090136
Lymphocyte percentage	0.094608
NLR ratio	0.080811
SGOT	0.145598
SGPT	0.106513
Serum creatinine	0.087070
Duration of MTX therapy*	0.070755
Initial MTX dose	0.006015
Initial MTX dose class	0.007228
Maximal MTX dose	0.017936
Maximal MTX dose class	0.008619
MTX monotherapy	0
MTX plus sulfasalazine	0.053353
MTX plus leflunomide	0.009805
MTX plus mycophenolate	0
MTX plus hydroxychloroquine	0.008593
Triple regimen	0

**Figure 2:**
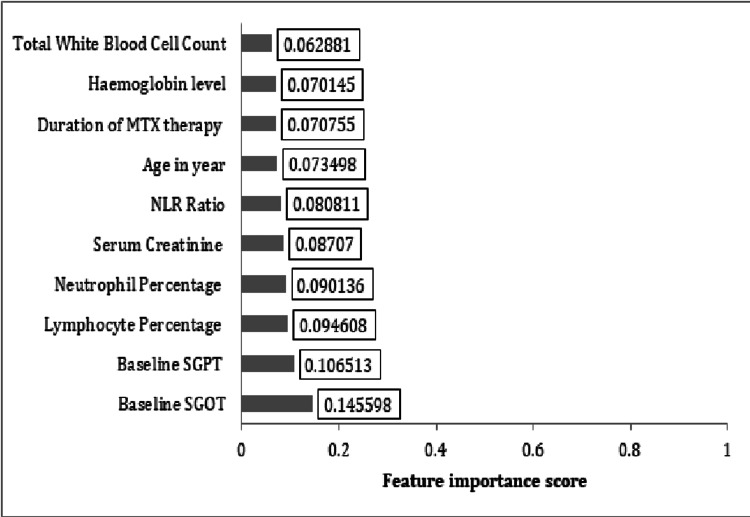
Top 10 feature importance of the full-feature ML classifier model for MTX-associated hepatic dysfunction MTX, methotrexate; NLR, neutrophil-to-lymphocyte ratio; SGOT, serum glutamic-oxaloacetic transaminase; SGPT, serum glutamate pyruvate transaminase; ML, machine learning

The top 10 features and their scores are shown graphically in Figure 2.

**Figure 3:**
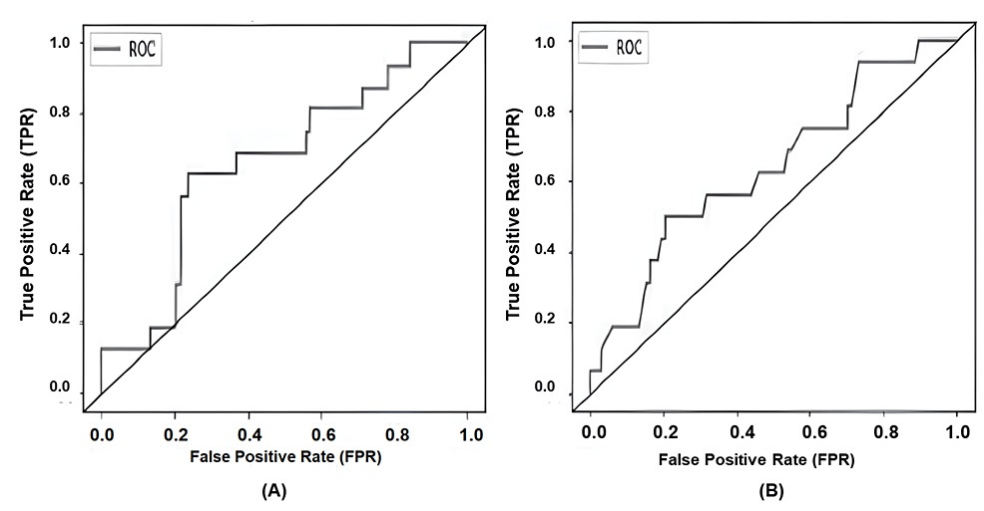
Comparison of ROC curves of liver enzyme elevation machine learning models: (A) complete feature vs. (B) limited feature ROC, receiver operating characteristic

From the top 10 features shown in Figure 2, a limited feature random classifier model was trained again. A precision of 0.81, recall of 0.86, and an accuracy/F1 score of 0.86 were achieved in this limited feature model. Figure 3 shows the AUC of the full-feature model versus the limited feature models, respectively (0.658 vs 0.635).

## Discussion

Our study highlights the feasibility of developing an ML model to predict the development of liver enzyme elevation in RA patients on treatment with MTX. We reviewed the EHRs of 569 patients with RA and extracted their clinical, biochemical, and prescription information for analysis. These data were then used to train a random forest model to predict the risk of liver enzyme elevation, which achieved an accuracy score of 0.87. Our analysis showed that the most important attributes that had the strongest influence on the development of hepatic dysfunction were white cell count along with the neutrophil and lymphocyte percentages, hemoglobin levels, duration of therapy, age, NLR ratio, creatinine, and baseline SGOT and SGPT levels. The limited feature model developed using the most important attributes also proved reliable, retaining much of the predictive accuracy.

As most of these parameters are readily available during a clinical visit, it is possible to easily apply these features during their first visit to predict the risk of a therapy-related adverse event. The current ACR guidelines recommend monthly monitoring of SGOT and SGPT for the first three months and then two to three monthly after that [5]. Using a prediction model, we could potentially stratify these patients based on the risk of therapy-related adverse events. Low-risk patients could be identified for less frequent testing and in-person visits, thus helping to cut costs. In contrast, high-risk patients could be identified for lower ceiling doses of MTX and planned for early tapering, thus minimizing the risk of liver damage.

Accurate risk prediction is a cornerstone of public health. Several risk prediction scores or tools have been developed to predict the risk of cardiovascular events and malignancies [21,22]. However, such individual risk assessment tools are lacking in autoimmune diseases.

Elevation of liver enzymes is the second most common adverse event noted in patients on treatment with MTX, with more than 20% reporting this event in a large meta-analysis of MTX monotherapy [23]. The risk of this iatrogenic adverse event is even higher in combination DMARD therapy patients [24]. Other studies also have shown similar risk factors for developing MTX-related hepatic dysfunction as identified by our full feature model. Baseline elevation of SGPT was identified as the strongest predictor of SGPT elevations during MTX therapy in a recent study of 213 RA patients followed up for a mean duration of 4.3 years [25].

Another important risk factor for the elevation of liver enzymes was the presence of non-alcoholic fatty liver disease (NAFLD) [19]. We excluded all ultrasounds conducted after the initiation of MTX; to avoid overfitting the model, as patients with elevated liver enzymes are more likely to have undergone liver biopsies. Studies have shown the role of NAFLD screening in predicting MTX associated with hepatic dysfunction [26]. As with all ML models, the greater the number of data points analyzed, the greater the accuracy of the model [27]. Therefore, inputting additional data (NAFLD status, genomic data) in future studies could lead to a more accurate model. Another limitation of our study was the population demographic sampled; as the model was developed using only patients of South Asian (Indian) descent, transferring this model to other populations may be difficult.

## Conclusions

We successfully developed a highly accurate ML model to predict the risk of liver enzyme elevation in RA patients on treatment with MTX. Similar models could help individualize treatment approaches and minimize therapy-related adverse events. Further prospective studies with expanded analysis including pharmacogenomic and other risk factors (such as NAFLD) involving larger and more diverse populations could develop more robust and clinically useful models.
